# A Prospective Interventional Study on LigaSure^TM^ Haemorrhoidectomy as a Daycare Procedure

**DOI:** 10.21315/mjms2021.28.5.10

**Published:** 2021-10-26

**Authors:** Abdel Latif K Elnaim, Shareef Musa, Michael Pak-Kai Wong, Ismail Sagap

**Affiliations:** 1 Department of Surgery, Kassala Police Hospital, Kassala, Sudan; 2 Department of Surgery, School of Medical Sciences, Universiti Sains Malaysia, Kubang Kerian, Kelantan, Malaysia; 3 Department of Surgery, Hospital Universiti Sains Malaysia, Kubang Kerian, Kelantan, Malaysia; 4 Department of Surgery, Universiti Kebangsaan Malaysia Medical Centre, Kuala Lumpur, Malaysia

**Keywords:** LigaSure, excisional haemorrhoidectomy, Milligan-Morgan, daycare surgery, regional proctology

## Abstract

**Objective:**

This study was designed as a prospective and interventional study that evaluated LigaSure™ haemorrhoidectomies with regional anaesthesia as a daycare procedure.

**Methods:**

Patients with third- and fourth-degree haemorrhoids were recruited from the clinic from January 2018 to December 2019. The procedure was performed as a day case under regional anaesthesia. Using a LigaSure^TM^ device, excisional haemorrhoidectomies (Milligan–Morgan haemorrhoidectomy) were performed without sutures or an anal sponge. We evaluated wound bleeding, pain and urinary retention per daycare protocols.

**Results:**

A total of 264 patients were enrolled. There were 153 males (57.9%) with a median age of 30 years old (range 16 years old–80 years old). A total of 142 patients (54%) had third-degree haemorrhoids, while the rest had fourth-degree haemorrhoids. The median operating time was 8 min (range 4 min–17 min) and minimal blood loss was observed. During follow-up, the complications were one case (0.3%) had anal stenosis, one case (0.3%) had minimal bleeding and one case (0.3%) had urine retention. Upon discharge, four patients (1.5%) required additional analgesia and another four (1.5%) developed post-spinal headaches. No incontinence was encountered.

**Conclusion:**

LigaSure™ excisional haemorrhoidectomy is a safe and effective daycare procedure with acceptable re-admission and complication rates.

## Introduction

Excisional haemorrhoidectomy is still the gold standard for the surgical treatment of haemorrhoid disease and has the lowest recurrence rate (1). A frequently performed procedure is the Milligan–Morgan excisional haemorrhoidectomy, which is an in-patient procedure that is performed electively (2). At the present time, this procedure is performed using diathermy, as it minimises sphincter injury while providing a clear surgical field for effective haemostasis. It also avoids the use of sutures and anal packs. However, this approach may cause severe post-operative pain, which requires more analgaesia and antibiotic use, such as metronidazole (3).

Post-operative pain has been one of the main concerns in the treatment of symptomatic haemorrhoids and there have been several treatment modifications for pain reduction that have enabled surgical interventions to be done on a daycare basis. Haemorrhoidectomy using LigaSure^TM^ is one such option and there are minimal complications, even among the senior population or those with pacemakers (4).

The LigaSure^TM^ device is a vessel-sealing system. It has gained popularity because it has superiority in achieving rapid haemostasis and replaced the use of sutures and clips. This advanced bipolar system is able to detect the type of tissue between the instrument jaws and delivers an appropriate amount of pressure and energy to the collagen and elastin within the vessel wall to seal it permanently with minimal collateral thermal damage. It also provides audio-feedback informing its operator the completion of the seal cycle (5). The use of the LigaSure^TM^ device was shown to be superior, especially when large vascularised tissue sealing was required during excisional haemorrhoidectomy of grade IV internal haemorrhoids (6).

This study evaluated the use of LigaSure^TM^ device in haemorrhoidectomy daycare procedures performed on patients with symptomatic third- and fourth-degree haemorrhoids.

## Methods

After obtaining approval from the local institutional review board, all patients diagnosed with third- and fourth-degree haemorrhoids at Kassala Police Hospital’s outpatient clinic in Sudan from January 2018 to December 2019 were invited to participate in the study. Convenient sampling was used in this study to recruit as many patients as possible. All patients with third- and fourth-degree haemorrhoids were offered only LigaSure^TM^ haemorrhoidectomy during this study period. However, they were screened for eligibility for daycare surgery and informed consent was obtained. Antibiotics including metronidazole were given 1 h prior to the operation. Patients underwent spinal anaesthesia or a saddle block and were in a lithotomy position for the procedure. Excisional haemorrhoidectomy (Milligan–Morgan technique) was performed using a LigaSure^TM^ device. The haemorrhoidal pedicles were sealed with a LigaSure^TM^ device only. The treatments were applied to all three columns in patients with third- and fourth-degree haemorrhoids. Haemostasis, whenever needed, was secured using diathermy. No sutures or anal sponge packing were used perioperatively. This procedure was performed by a single surgeon to reduce operator bias in the study. The patients were observed until they completely recovered from spinal anaesthesia and the wound was monitored for bleeding. Pain scores and urinary retention were evaluated upon discharge. Discharge on the day of surgery was planned for all patients once they recovered from spinal anaesthesia and had no bleeding, had tolerable pain and were able to urinate spontaneously. They were all discharged with seven days of medications, including 1 g paracetamol (twice a day), 75 mg diclofenac sodium (once a day or as required) and 500 mg metronidazole (twice a day).

The measured outcomes were intra-operative bleeding, reactive bleeding, pain control, need for prolonged analgesia, readmission rate, anal stenosis and anal incontinence. Intraoperative bleeding was measured by recording the amount of soaked gauze and cases were subdivided into quarter staining, half staining and full staining. Patients were also interviewed regarding their levels of satisfaction with the outcome of the operation at two weeks post-operation. The options for evaluations included very satisfactory, satisfactory and unsatisfactory. During their weekly follow-up, patients were also asked about their return to normal daily activity. The numerical data were presented as medians with an interquartile range, and the categorical data were presented as frequencies and percentages.

## Results

A total of 264 patients were enrolled in this study (Table 1), including 154 males (58.3%) and 110 females (41.7%), with a median age of 30 years old (range 16 years old–80 years old). The study recruited 131 patients (49.6%) with third-degree haemorrhoids, whereas fourth-degree haemorrhoids were found in 133 patients (50.4%). The median operating time was 8 min (range 4 min–17 min) with minimal blood loss staining approximately one-quarter of a gauze, which was less than 5 mL, in 256 patients (97%). For post-operative pain assessment, the minimum score on the visual analogue scale (VAS) was 3 and the median discharge time was 6 h after the procedure.

Post-operative complications (Table 2) were anal stenosis in one patient (0.4%), reactive bleeding in one patient (0.3%) and urinary retention in two patients (0.8%). Three patients (1.1%) required additional analgaesia upon discharge. Four patients (1.5%) developed post-spinal headache, which was detected upon discharge and treated to an immediate effect with conservative medical measures. There was no report of anal incontinence during the follow-up.

Two patients, however, were readmitted within one week for upper gastrointestinal bleeding (UGIB) that was not related to the procedure. It was associated with non-steroidal anti-inflammatory drugs (NSAIDs), evidenced by the multiple antral erosions on endoscopic examination and treated with intravenous proton pump inhibitors.

The patient with post-operative anal stenosis was treated conservatively with stool softener and fibre diet modification. The symptoms eventually improved without any additional procedures.

As for those with acute urinary retention, we performed clean intermittent urinary catheterisation with bladder drainage once and it resolved without any recurrence before discharge. None were readmitted for acute urinary retention after discharge.

The patient-reported level of satisfaction (Table 3) was high (very satisfied) in 237 patients (89.8%), moderate (satisfied) in 25 patients (9.5%) and only two patients (0.8%) were unsatisfied in this cohort. More than 87% of the patients had returned to their daily activities by the second week after their operation.

## Discussion

The LigaSure^TM^ device offers promising haemostatic control, thereby negating the need for pedicle ligation in haemorrhoidectomies. Improved haemostasis may offer better visibility and, hence, accurate dissection. This approach has been found to be superior to conventional scissors or cauterised haemorrhoidectomies in terms of shorter operative times, less pain, and a lower incidence of urinary retention (7). Apart from these results, it was also shown to produce earlier wound healing and a shorter time to return to work (7).

The daycare protocol was designed to shorten the hospital stay and allow early return to work. This could reduce hospital-acquired infections and other post-operative complications, while also bringing down the overall treatment cost (8).

This prospective study confirmed that LigaSure^TM^ haemorrhoidectomy could be performed quickly, with a median time of 8 min and adequate haemostasis. These findings are consistent with those reported in the literature (7, 9–10).

Post-operative pain remains a major concern for patients undergoing haemorrhoid treatment. Many modifications to the standard haemorrhoidectomy have been made by incorporating the use of energy with cautery devices, ultrasonic technology and LigaSure^TM^. With these changes, a daycare approach seems reasonable if pain is not a significant obstacle to overcome (4). In our study, most of patients demonstrated a median VAS pain score of 3 and the score improved over time without further complications. Furthermore, the use of spinal anaesthesia in our daycare surgery has had a negligible complication rate and was shown to be highly effective for perioperative pain control (11). In this study, all patients received metronidazole, which was proven to improve pain control, patient satisfaction and an earlier return to normal activity, which reproduced the results reported in a previous report (3).

Our study also revealed that the use of spinal anaesthesia was useful in providing immediate post-operative pain relief in haemorrhoidectomies as daycare cases with minimal related complications. Of note, when using regional block techniques, longer observation times might be needed, especially for urinary retention and headaches. We found that the majority of our patients were successfully discharged after 6 h on the same day of surgery once the regional anaesthesia wore off. Longer stays from regional blocks were not observed in our cohort as the majority of patients were able to recover within 6 h on the same day (12). Therefore, it may be sensible to expect readmissions due to delayed presentations of complications from spinal anaesthesia, such as spinal headaches, when the procedures are being performed as daycare cases.

In our study, two patients were readmitted for NSAID-induced UGIB. These cases of bleeding could also be attributed to the NSAIDs used for pain after surgery, evidenced by multiple antral erosions on endoscopic examination.

A limitation of our study is the prospective design. A superior approach would be to randomise the LigaSure^TM^ with conventional scissors or diathermy Milligan–Morgan haemorrhoidectomy at a daycare procedure setting and compare their outcomes. As the patient-reported satisfactory response was recorded with an interview, there is potential bias to the response. This study’s strength was the minimal selection bias, as all patients who fulfilled the criteria within the study period were invited to participate. In our cohort, there was no recurrence. This is coherent with the available literature on excisional haemorrhoidectomy. Our follow-up period was short, which may also contribute to the low recurrence rate. The most important feature was selecting suitability and compliance with the daycare protocol, which ensured the good outcome of the study.

## Conclusion

LigaSure^TM^ excisional haemorrhoidectomy under regional anaesthesia is a safe and effective daycare procedure with acceptable readmission and complication rates. It is an easy, reproducible and quick procedure for haemorrhoidectomies, especially in developing countries such as Sudan.

## Figures and Tables

**Table 1:** Demographic and perioperative data

Sociodemography	
Age median (years old)	30 (16–80)
Gender	
Male	154 (58.3%)
Female	110 (41.7%)
Type of haemorrhoid	
Grade III	131 (49.6%)
Grade IV	133 (50.4%)
Operation time (min)	8 (4–17)
Blood loss	
¼ Stained gauze	256 (97.0%)
½ Stained gauze	8 (3.0%)
Pain score (VAS)	3 (2–8)
Time to discharge (hours)	6 (4–8)

**Table 2:** Complications and short-term outcomes

Immediate/Early complications	
Urinary retention	2 (0.8%)
Persistent pain (> 2 weeks)	3 (1.1%)
Bleeding	1 (0.4%)
Spinal anaesthesia related complications	4 (1.5%)
Late complications	
Anal stenosis	1 (0.4%)
Anal incontinence	0
Readmission	
NSAID-related upper GI bleed	2 (0.1%)
Outcome at 6 months	
Early symptomatic haemorrhoid recurrence at 6 months	0
Anal incontinence at 6 months	0

**Table 3:** Patient satisfaction and return to daily activity

Patient’s satisfaction (within 2 weeks)	
Very satisfied	237 (89.8%)
Satisfied	25 (9.1%)
Not satisfied	2 (1.1%)
Return to daily activity	
At 1 week	22 (8.3%)
At 2 weeks	230 (87.1%)
At 3 weeks	264 (100%)
